# Malnutrition Screening Tools for Geriatric Patients Presenting to Emergency Department: Agreement and Prognostic Utility for Hospital Admission and Length of Stay

**DOI:** 10.3390/healthcare14111488

**Published:** 2026-05-27

**Authors:** Ali Halıcı, Ezgi Cesur

**Affiliations:** 1 Department of Emergency Medicine, Faculty of Medicine, Kütahya Health Sciences University, Kütahya 43000, Türkiye; 2 Department of Emergency Medicine, Kütahya State Hospital, Kütahya 43000, Türkiye; drezgiyagyemez@gmail.com

**Keywords:** malnutrition, frailty, emergency department, geriatric patients, MUST, NRS-2002, Subjective Global Assessment, length of stay

## Abstract

**Background:** Malnutrition is a major global geriatric health problem, reported in approximately one-fifth of older adults worldwide and occurring even more frequently in acute care and hospital settings. Among older adults presenting to the emergency department (ED), nutritional vulnerability is often underrecognized because early ED decision-making is primarily dominated by acute physiological instability and the need for rapid disposition. Clarifying whether commonly used malnutrition screening tools provide clinically useful information beyond frailty, comorbidity burden and acute illness severity may help determine their role in early geriatric ED risk stratification, in-hospital care planning, and resource utilization. **Objectives:** To evaluate the prevalence, agreement, and clinical utility of three validated malnutrition screening tools the Malnutrition Universal Screening Tool (MUST), Nutritional Risk Screening 2002 (NRS-2002), and the clinician-administered Subjective Global Assessment (SGA) in older ED patients, and to examine their associations with hospital admission and length of stay (LOS). **Methods:** This prospective single-center study included 325 patients aged ≥65 years presenting to the ED. Nutritional status was assessed using the MUST, the NRS-2002, and the SGA. Agreement between tools was evaluated using Cohen’s kappa, positive percent agreement, and negative percent agreement. Associations with hospital admission were analyzed using multivariable logistic regression adjusted for age, sex, Clinical Frailty Scale, National Early Warning Score 2, and Charlson Comorbidity Index. Multivariable linear regression was used to identify predictors of LOS. **Results:** Overall, 32.6% of patients required hospital admission. Among admitted patients, the median hospital length of stay was 5 days (IQR 2–9). The prevalence of high nutritional risk varied substantially across tools, from 16.6% with the MUST to 41.5% with the NRS-2002 and 23.4% with the SGA. Agreement between tools was moderate overall (κ = 0.41–0.60), with moderate concordance in identifying low-risk and high-risk patients. After adjustment for clinically relevant covariates, none of the screening tools was independently associated with hospital admission. However, high-risk classification by the NRS-2002 was independently associated with prolonged LOS (β = 0.47, 95% CI 0.10–0.85; *p* = 0.01), whereas the MUST and the SGA were not. **Conclusions:** In older ED patients, malnutrition screening tools did not add independent value for predicting immediate hospital admission beyond frailty, comorbidity burden, and acute illness severity. However, the NRS-2002 was associated with longer hospital stay, suggesting potential value for early identification of patients who may require more complex in-hospital care and resource planning.

## 1. Introduction

The rapid shift in global demographics toward an aging population has led to a marked increase in the clinical complexity of patients presenting to the ED. In this setting, clinical decision-making is predominantly focused on acute physiological derangements and immediately life-threatening conditions. As a consequence, chronic yet clinically significant problems such as malnutrition may be overlooked. Malnutrition is common among geriatric patients but remains underrecognized in routine clinical practice, despite being widely acknowledged as a major public health concern worldwide [1,2]. In the emergency department context, previous studies have reported a high prevalence of malnutrition among geriatric patients, with considerable variability depending on the screening tool used [3,4,5].

From a global perspective, malnutrition in older adults represents a substantial and growing healthcare problem. Previous multinational evidence suggests that approximately one fifth of older adults worldwide are affected by malnutrition, while prevalence is even higher in acute care and hospital-based populations [1,6]. This worldwide burden is particularly relevant for emergency departments, where older adults frequently present with acute illness superimposed on frailty, multimorbidity, and functional vulnerability. Therefore, identifying nutritional risk during the early ED encounter may have implications not only for individual patient care but also for hospital resource use and discharge planning. In older adults, malnutrition often develops through the complex interaction of age-related physiological and metabolic changes. Reduced appetite (anorexia of aging), impaired taste and smell, decreased gastrointestinal absorption, chronic low-grade inflammation, sarcopenia and polypharmacy may all contribute to increased nutritional vulnerability. In addition, cognitive impairment, depression, poor dentition and social isolation frequently lead to inadequate nutritional intake and progressive functional decline. These multifactorial mechanisms make nutritional assessment particularly challenging in geriatric emergency populations [7,8].

In aging populations, malnutrition develops insidiously through the interaction of multiple factors, including chronic disease burden, age-related physiological changes, polypharmacy, cognitive impairment and social vulnerability [9,10]. These factors render geriatric patients particularly susceptible to nutritional deterioration during episodes of acute illness. The emergency department, often representing the first point of contact with the healthcare system during such episodes, therefore provides a critical opportunity for the early identification of nutritional risk.

A growing body of evidence has demonstrated significant associations between malnutrition or nutritional risk and adverse outcomes, including prolonged hospital length of stay, increased complication rates, functional decline, institutionalization and mortality [11,12,13]. However, the impact of malnutrition on early emergency care outcomes remains less clearly established. Admission decisions in the ED are largely driven by acute illness severity and physiological instability and it is uncertain whether nutritional vulnerability independently influences this process. One of the main challenges in addressing this question is the lack of consensus regarding the most appropriate malnutrition screening tool for use in acute care environments.

Several validated instruments are currently used to assess malnutrition risk, including the MUST, the NRS-2002 and the SGA. These tools differ substantially in their underlying conceptual frameworks [14,15]. The MUST primarily relies on anthropometric measurements and recent weight loss, whereas the NRS-2002 incorporates disease severity and an age-adjusted component, offering a more comprehensive evaluation of nutritional risk in acutely ill patients. In contrast, the SGA is based on clinical history and physical examination findings, providing a more subjective yet holistic assessment. Owing to these structural differences, the application of these tools, particularly in acute care settings such as the ED, may result in the classification of different patient subgroups as being at nutritional risk.

In this clinical scenario, older ED patients frequently present with overlapping acute illness, frailty, comorbidity and functional vulnerability, making risk stratification challenging. Although malnutrition screening is recommended in many healthcare settings, its practical value during early ED decision-making remains uncertain. In particular, it is unclear whether nutritional screening tools contribute independent information beyond established indicators of acute illness severity, frailty and comorbidity burden or whether they primarily identify patients who may require longer and more complex in hospital care after admission. Addressing this gap is important for determining how nutritional screening should be integrated into geriatric emergency care pathways. Therefore, the present study aimed to evaluate the prevalence and agreement of the MUST, the NRS-2002 and the SGA in older ED patients and to investigate their associations with hospital admission and length of stay.

## 2. Materials and Methods

### 2.1. Study Design and Setting

We conducted a single-center, prospective observational study in the ED of Kutahya Health Science University, a tertiary care teaching hospital in Türkiye. The study was designed to evaluate malnutrition risk in geriatric ED patients using three established nutritional screening tools and to examine the association between malnutrition risk classifications and ED-related clinical outcomes. The age threshold of 65 years was selected in accordance with the most widely accepted definition of older adulthood used in geriatric research and international epidemiological studies.

### 2.2. Study Population and Participant Selection

Consecutive patients aged ≥65 years presenting to the ED during the study period (1 September 2025 to 1 January 2026) were screened for eligibility. Patients were included if they (1) were ≥65 years, (2) underwent standardized ED assessment enabling calculation of predefined clinical severity/frailty/comorbidity scores and (3) had sufficient information to complete nutritional screening assessments.

**Figure 1:**
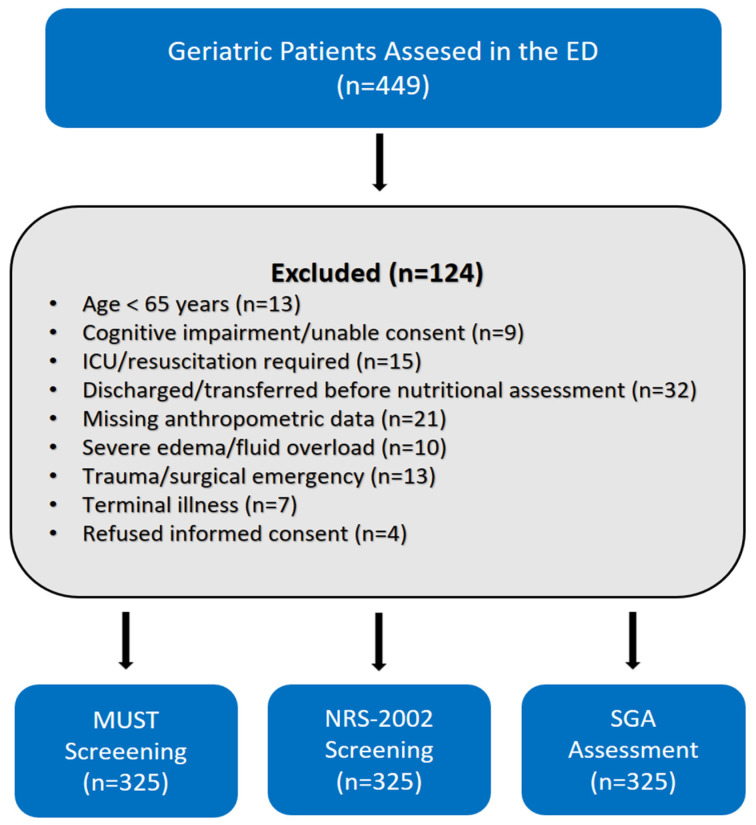
Study flow diagram of patient inclusion and exclusion.

Patients were excluded if key data elements required for nutritional tool scoring or outcome ascertainment were unavailable, if participation was declined when consent procedures were required by the institutional review board, or if they had terminal palliative conditions, were receiving active oncological treatment, or were permanently immobilized/bedridden. Permanently immobilized or bedridden patients were excluded because reliable anthropometric assessment and standardized functional evaluation could not be ensured in this subgroup. A flow chart illustrating the patient selection process, including eligibility assessment and reasons for exclusion, is provided in Figure 1.

### 2.3. Data Collection Procedures

Data were collected prospectively using a standardized case report form by trained study personnel (emergency physicians and/or research staff) during the ED encounter. Demographics (age, sex), presenting complaint, and routine clinical variables were recorded from direct patient evaluation and the electronic medical record. For each participant, clinical severity and baseline risk characteristics were captured at the earliest feasible time point during ED care (typically during triage or initial physician assessment) to reflect presentation status.

### 2.4. Anthropometric Measurements (Height and Weight in the ED)

Because anthropometric data are central to malnutrition screening, a standardized and pragmatic ED protocol was used to obtain height and weight with maximal feasibility and internal consistency. Body weight and height were recorded at the earliest feasible time during the ED encounter.

### 2.5. Ambulatory Patients

Body weight was measured using a calibrated digital scale in the ED. Patients were asked to remove shoes and heavy outer garments when possible. Height was measured in the standing position using a stadiometer (or wall-mounted height measure), with the head positioned in the Frankfurt plane when feasible.

### 2.6. Non-Ambulatory or Clinically Unstable Patients

For patients who could not stand safely (e.g., mobility limitation, marked dyspnea, hemodynamic instability or sedation), direct standing measurements were not forced. In such cases, height and/or weight were obtained using a hierarchical approach:

(a) The most recent documented height/weight available in the hospital electronic record, if judged clinically plausible;
(b) Patient or caregiver report of usual or recent weight/height when documented measurement was not available;
(c) When neither source was reliable, anthropometric data were recorded as missing and the patient was excluded from analyses requiring those variables, per the a priori plan.

Patients who were permanently immobilized or bedridden were excluded because reliable standardized anthropometric assessment and functional evaluation could not be ensured in this subgroup.

Body mass index (BMI) was calculated as weight (kg) divided by height squared (m^2^). BMI categories were defined as ≤18.5, 18.6–24.9, 25.0–29.9, and ≥30.0 kg/m^2^.

### 2.7. Clinical Scoring Systems

To characterize acute illness severity, frailty and comorbidity burden, NEWS2, CFS and CCI were calculated for all participants using standard definitions. These scores were selected because they represent complementary domains relevant to geriatric emergency care: acute physiological derangement, baseline functional vulnerability and chronic disease burden.

NEWS2 was used to quantify acute physiological severity at presentation. The score was calculated using routinely recorded ED parameters, including respiratory rate, oxygen saturation, need for supplemental oxygen, systolic blood pressure, heart rate, body temperature and level of consciousness. The earliest available measurements obtained during triage or initial physician assessment were used to reflect the patient’s initial ED condition.

CFS was used to assess frailty based on the patient’s baseline functional status before the acute illness. CFS classification was determined using clinical judgment supported by information from the patient, caregiver and medical records when necessary. For descriptive analyses, CFS categories were grouped as CFS 1–3, CFS 4, and CFS ≥ 5.

CCI was used to quantify comorbidity burden. The score was calculated from documented chronic diagnoses in the electronic medical record according to the standard Charlson Comorbidity Index scoring system.

Clinical severity, frailty and comorbidity variables required for NEWS2, CFS and CCI were obtained from the standardized case report form and electronic medical records. The same assessment procedure was applied to all participants to improve consistency.

### 2.8. Nutritional Screening Tools and Definitions

The three nutritional assessment tools were selected a priori because they are established, widely used, and clinically feasible instruments that represent different conceptual approaches to malnutrition assessment. The MUST was included as an anthropometry- and weight loss-based screening tool, the NRS-2002 was included because it incorporates nutritional impairment together with disease severity and age, and the SGA was included as a clinician-administered global assessment based on history and physical examination. Therefore, the use of these three tools allowed for comparison of complementary approaches rather than evaluation of a single screening construct. To ensure standardization, all tools were applied according to their original scoring algorithms and predefined cut-off values. No local recalibration of thresholds was performed, because the aim of the study was to evaluate agreement and prognostic utility of commonly used validated tools in an ED population, not to derive a new prediction model.

No new questionnaire or study-specific nutritional screening scale was developed for this study. A standardized case report form was used only to prospectively record demographic, anthropometric, clinical and nutritional variables required for the study assessments. Nutritional status was evaluated using three previously validated and widely used tools: the MUST, the NRS-2002 and the SGA. These tools were applied according to their original scoring principles and predefined cut-off values.

The MUST was calculated using body mass index, unintentional weight loss during the previous 3–6 months and the acute disease effect. BMI was scored according to standard MUST thresholds, weight loss was scored according to the percentage of recent unintentional weight loss and an acute disease effect score was added when applicable. A total MUST score of 0 indicated low risk, 1 indicated medium risk and ≥2 indicated high malnutrition risk.

The NRS-2002 was calculated by combining nutritional impairment and disease severity scores. Nutritional impairment was assessed using BMI, recent weight loss and reduced oral intake, while disease severity reflected the expected increase in nutritional requirements due to acute illness. One additional point was added for patients aged ≥70 years according to the standard NRS-2002 algorithm. A total NRS-2002 score ≥3 was defined as high nutritional risk.

The SGA was performed using clinical history and physical examination findings, including recent weight loss, dietary intake changes, gastrointestinal symptoms, functional capacity, metabolic stress related to underlying disease, loss of subcutaneous fat, muscle wasting and edema or ascites. Patients were classified as SGA A, B or C. For the present analysis, SGA A was considered well-nourished, whereas SGA B or C was defined as malnourished.

For cross tool comparisons, classifications were harmonized into a binary format as high-risk/malnourished versus not high-risk based on these predefined thresholds.

### 2.9. Outcomes

The primary outcome was hospital admission from the ED (admitted vs. discharged).

The secondary outcome was LOS among admitted patients, defined as the number of days from hospital admission to discharge, derived from administrative/electronic records.

### 2.10. Statistical Analysis

All statistical analyses were performed using IBM SPSS Statistics version 27.0 (IBM Corp., Armonk, NY, USA), R software version 4.4.1 (R Foundation for Statistical Computing, Vienna, Austria) and Python (version 3.12.7, Anaconda Inc.).

Based on an expected hospital admission rate of 40% derived from previous studies of geriatric emergency department populations, a minimum sample size of 305 patients was estimated using a 95% confidence level and a 5.5% margin of error. Accordingly, the final sample of 325 patients was considered sufficient for the primary analysis.

Continuous variables were assessed for normality using visual inspection of histograms and the Shapiro–Wilk test. Normally distributed variables were presented as mean ± standard deviation, whereas non normally distributed variables were summarized as median (interquartile range, IQR). Categorical variables were expressed as counts and percentages. Baseline demographic and clinical characteristics were compared between patients requiring hospital admission and those discharged from the emergency department. For continuous variables, comparisons were performed using the independent samples *t* test or the Mann–Whitney U test, as appropriate. Categorical variables were compared using the chi square test or Fisher’s exact test when expected cell counts were small.

Nutritional status was evaluated using three validated malnutrition screening tools: the MUST, the NRS-2002 and the SGA. High nutritional risk was defined as MUST ≥ 2, NRS-2002 ≥ 3 and SGA class B/C, consistent with established criteria. Agreement among the three screening tools was assessed using Cohen’s kappa (κ) statistic, along with overall agreement rates. In addition, positive percent agreement (PPA) and negative percent agreement (NPA) were calculated to further characterize concordance in identifying patients with and without malnutrition risk.

To examine the association between malnutrition risk and hospital admission, univariable analyses were initially performed, followed by multivariable logistic regression models with hospital admission as the dependent outcome. Each malnutrition screening tool was entered separately into an identical base model to avoid multicollinearity. Covariates included age, sex, CFS, NEWS2 score and CCI. Results were reported as odds ratios (ORs) with 95% confidence intervals (CIs).

Given the absence of an independent association with hospital admission, secondary analyses evaluated whether malnutrition screening tools were associated with hospital LOS. Multivariable linear regression analyses were conducted with LOS as the dependent variable, adjusting for the same clinically relevant covariates (age, sex, CFS, NEWS2 and CCI). Regression coefficients (β) were presented with corresponding 95% CIs.

All statistical tests were two-tailed, and a *p* value < 0.05 was considered statistically significant.

## 3. Results

A total of 325 patients aged ≥65 years presenting to the emergency department were included in the analysis. The mean age of the cohort was 76.4 ± 7.2 years and 52% (*n* = 169) were female. Overall, 106 patients (32.6%) required hospital admission, while 219 patients (67.4%) were discharged from the emergency department. In crude comparisons, hospitalized and non-hospitalized patients were similar with respect to age, sex distribution, body weight, height, BMI categories, CCI, and CFS categories (all *p* > 0.05). In contrast, NEWS2 scores were significantly higher in patients requiring hospital admission compared with those discharged (7.7 ± 2.2 vs. 7.1 ± 1.9, *p* = 0.02). Baseline demographic and clinical characteristics stratified by hospital admission status are summarized in Table 1.

**Figure 2:**
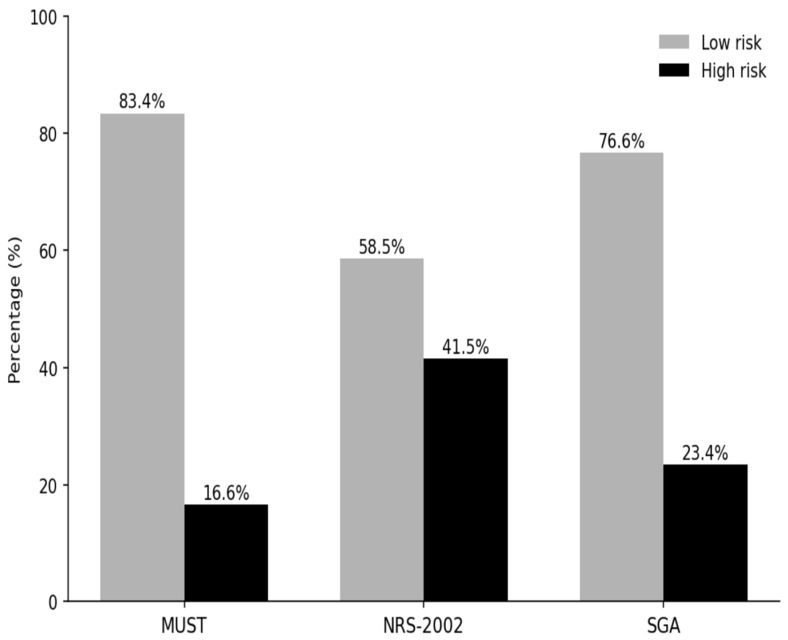
Proportion of patients classified as being at nutritional risk according to MUST, NRS-2002 and SGA (risk defined as MUST ≥ 2, NRS-2002 ≥ 3 and SGA class B/C). MUST: Malnutrition Universal Screening Tool, NRS-2002: Nutritional Risk Screening 2002, SGA: Subjective Global Assessment.

All patients included in the study were assessed for nutritional status using three validated malnutrition screening tools. According to the MUST, 16.6% of patients were classified as being at high risk for malnutrition, while an additional 12.0% were identified as having a moderate risk. Using the NRS-2002, 41.5% of the study population was categorized as being at high nutritional risk. Assessment with the Subjective Global Assessment (SGA) revealed that 23.4% of patients were classified as malnourished, including 19.1% with moderate malnutrition and 4.3% with severe malnutrition (Figure 2).

**Figure 3:**
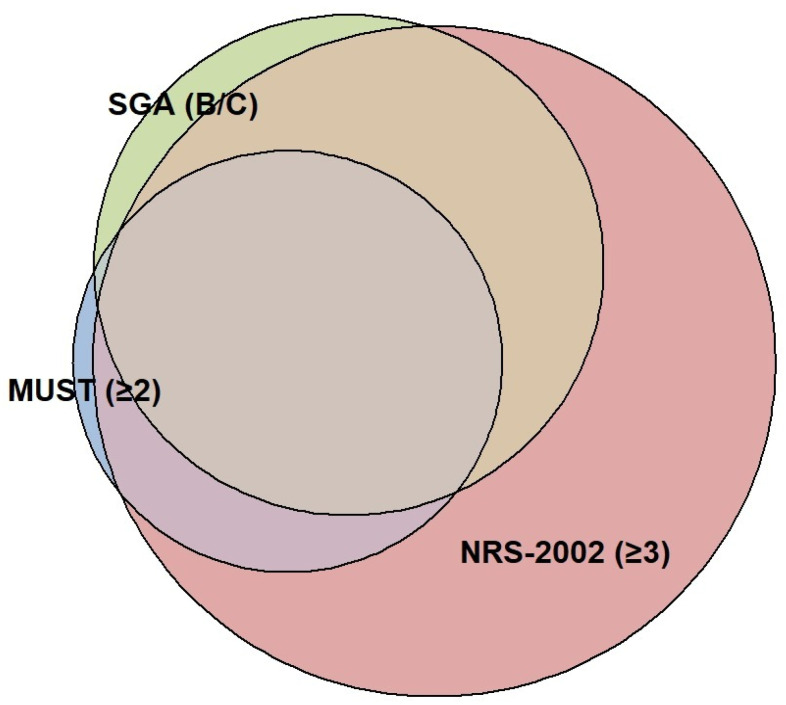
Overlap of malnutrition risk classification by MUST, NRS-2002 and SGA.

Agreement analyses were performed to evaluate concordance among the three malnutrition screening tools. Overall, the analyses demonstrated moderate agreement between the tools. The agreement between the MUST and the NRS-2002 was moderate (agreement 73.8%, κ = 0.41), while the NRS-2002 and the SGA also showed moderate concordance (agreement 78.2%, κ = 0.52). Comparisons between the MUST and the SGA revealed the highest level of agreement, with an agreement rate of 87.1% and a Cohen’s κ value of 0.60, corresponding to the upper limit of moderate agreement. The consistently higher NPA values compared with positive percent agreement (PPA) indicate greater concordance among the screening tools in identifying patients without malnutrition risk, as opposed to those classified as being at high risk for malnutrition (Table 2 and Figure 3).

When hospital admission status was examined according to malnutrition screening tools, no significant association was observed between hospital admission and malnutrition risk as classified by the MUST, the NRS-2002 or the SGA (all *p* > 0.05) (Table 3). The proportions of patients categorized as high-risk by each screening tool were comparable between admitted and non-admitted patients.

Although CCI and CFS categories were similar in crude comparisons, multivariable logistic regression showed that higher CFS was independently associated with hospital admission (Table 4).

Given the absence of an association with the likelihood of hospital admission, we subsequently investigated whether malnutrition screening tools were associated with hospital length of stay (LOS). In multivariable linear regression analyses evaluating the association between malnutrition screening tools and LOS, age was consistently and inversely associated with LOS across all models, indicating shorter hospital stays with increasing age (Table 5). CFS scores were positively associated with LOS in Model A (MUST) and Model C (SGA), while this association did not reach statistical significance in Model B (NRS-2002). Similarly, higher CCI scores were independently associated with longer LOS across all models. NEWS2 score and sex were not independently associated with LOS in any of the models.

Among the malnutrition screening tools, NRS-2002 high-risk classification was independently associated with prolonged LOS after adjustment for confounding variables (β = 0.47, 95% CI 0.10–0.85, *p* = 0.01). In contrast, MUST high-risk status was not associated with LOS, and SGA B/C classification showed only a non-significant trend toward longer LOS.

## 4. Discussion

This study was designed to address whether commonly used malnutrition screening tools provide clinically meaningful information during the early evaluation of older adults in the emergency department, where decisions are often driven by acute physiological instability, frailty, and comorbidity burden. The main findings can be summarized as follows: first, the prevalence of nutritional risk varied substantially according to the screening tool used, with the NRS-2002 identifying the highest proportion of at-risk patients; second, agreement between the MUST, the NRS-2002, and the SGA was only moderate, indicating that these tools are not interchangeable in geriatric ED populations; third, none of the tools independently predicted hospital admission after adjustment for clinical severity, frailty, and comorbidity; and fourth, the NRS-2002 was independently associated with longer hospital length of stay. These findings suggest that malnutrition screening may have limited value for immediate admission decisions but may still contribute to early recognition of patients requiring more complex in-hospital care [16].

In our cohort, hospitalized and discharged patients were similar in terms of chronological age, sex distribution, anthropometric measurements, and BMI categories. This finding supports the concept that chronological age alone is insufficient for acute admission decisions in geriatric emergency care, where biological age, frailty, and acute physiological status may be more informative [17]. Similarly, BMI categories were not associated with hospitalization, suggesting that BMI alone may be an inadequate marker in older adults because it may fail to capture malnutrition risk, sarcopenia, and body composition changes [18,19].

The CCI and CFS are widely used in older populations to reflect comorbidity burden and frailty [20,21,22]. In the present study, crude comparisons between admitted and discharged patients did not show statistically significant differences in CCI or CFS categories. However, after adjustment for other clinically relevant variables in multivariable logistic regression, higher frailty as measured by the CFS was independently associated with hospital admission, whereas the association with CCI was weaker and less consistent across models. This apparent difference is not contradictory, but rather reflects the distinction between unadjusted group comparisons and adjusted analyses. Frailty may not be fully captured in simple between-group comparisons, yet its independent contribution to ED disposition decisions becomes evident when evaluated together with age, sex, acute illness severity, and comorbidity burden. In contrast, NEWS2 remained a more consistent predictor of hospital admission, supporting the view that ED disposition is primarily driven by acute physiological severity, with frailty providing additional prognostic context [23,24].

In our geriatric ED cohort, the prevalence of nutritional risk varied substantially according to the screening tool used: 16.6% with the MUST, 23.4% with the SGA, and 41.5% with the NRS-2002. This variability is expected because the instruments assess different domains of nutritional vulnerability and use different thresholds. The NRS-2002 identified the highest proportion of at-risk patients, likely because it incorporates disease severity and an age-adjusted component, which may be particularly relevant in acutely ill older ED patients. Similar findings have been reported in previous studies comparing nutritional screening tools in acute care and hospitalized populations [25,26,27]. In contrast, the MUST relies mainly on BMI, recent weight loss, and acute disease effect, while the SGA is based on clinical history and physical examination, which may explain their lower or intermediate risk estimates. The relatively high NEWS2 scores observed in both admitted and discharged patients may reflect the high baseline burden of frailty, comorbidity, and chronic physiological abnormalities in older ED patients, as well as acute but manageable conditions that do not necessarily require hospitalization.

In this study, agreement analyses demonstrated a moderate concordance among the three malnutrition screening tools. The highest agreement was observed between the MUST and the SGA (κ = 0.60), followed by NRS-2002-SGA and MUST-NRS-2002 (κ = 0.52 and κ = 0.41, respectively). These findings indicate that although the instruments are related, they cannot be used interchangeably, as they classify malnutrition risk differently in geriatric emergency department patients. In line with our results, Chávez Tostado et al. reported moderate agreement between the NRS-2002 and the SGA (κ = 0.52), while Velasco et al. observed moderate to substantial agreement between the MUST and the SGA (κ = 0.63) [28,29]. Thus, our findings are well-supported by the existing literature.

One of the clearest observations from our analysis was that the NPA values (ranging from 81.6% to 91.9%) were markedly higher than the PPA values. Clinically, this suggests that when one tool classifies a patient as “low-risk” (well-nourished), the other tools are highly likely to reach the same conclusion. In other words, there is strong consensus among these instruments in identifying patients with adequate nutritional status. However, this agreement diminishes when patients are categorized as “high-risk,” as reflected by the relatively low PPA values. Notably, a substantial proportion of patients identified as high-risk by the NRS-2002 were classified as “normal” by the MUST or the SGA, potentially leading to under-recognition of nutritional vulnerability. The fact that the NRS-2002 detected the highest proportion of at-risk patients in the overall cohort (41.5%) may be explained by its inclusion of acute illness-related stress in the scoring system, which may be particularly relevant in the emergency department setting. Therefore, in emergency department settings where acute illness severity and metabolic stress are prominent, screening tools such as the NRS-2002 that incorporate disease burden may be more suitable for identifying vulnerable geriatric patients who might otherwise be overlooked by purely anthropometric or more subjective assessments.

In our study, malnutrition screening tools did not show a significant effect on the likelihood of hospital admission among geriatric emergency department patients. The proportions of patients classified as high-risk according to all three screening instruments were similar between those who were admitted and those who were discharged. Both univariable analyses and multivariable logistic regression models adjusted for clinically relevant covariates demonstrated that none of the screening tools independently predicted hospitalization. The main factors associated with hospital admission were age (*p* < 0.001), NEWS2 score (*p* = 0.003) and frailty as measured by the CFS (*p* < 0.001). These findings indicate that admission decisions in the emergency department are primarily driven by the patient’s acute clinical condition and functional reserve rather than chronic nutritional status. Accordingly, malnutrition does not appear to serve as a marker for the immediate decision to hospitalize in this setting.

Given the limited ability of malnutrition screening tools to predict hospital admission, we further explored whether they were associated with more sensitive clinical outcomes, such as LOS. Among hospitalized patients, increasing age was inversely associated with LOS, while higher comorbidity burden was consistently associated with longer LOS. Frailty was associated with longer LOS in two of the three models. This suggests that while malnutrition screening may not influence immediate admission decisions in the emergency department, it may still provide important prognostic information by identifying patients at risk for poorer in hospital trajectories, including prolonged hospitalization and increased healthcare needs.

A noteworthy finding of our study was the consistent inverse association between age and LOS across all models, indicating that LOS decreased as age increased. The inverse association between age and LOS may appear paradoxical. Because mortality and discharge destination were not evaluated in this study, this finding should be interpreted cautiously. Possible explanations may include differences in clinical decision-making, discharge pathways, care goals, or unmeasured factors in the oldest patients. Similar heterogeneous age-related patterns have been reported in geriatric ED cohorts; for example, Launay et al. found that patients aged ≥85 years did not have a significantly increased risk of prolonged hospitalization compared with those aged 70–75 years [30]. Further studies including mortality and post-discharge outcomes are needed to clarify this relationship.

In addition, the significant association between frailty (CFS) and LOS in the MUST and SGA models highlights that frail patients are more vulnerable to in-hospital complications and often require longer and more complex care. However, the attenuation of this effect in the NRS-2002 model suggests that part of the frailty-related impact may be captured through nutritional risk, emphasizing the substantial overlap between malnutrition and frailty. Our finding that higher frailty scores were associated with prolonged LOS is consistent with previous studies [31,32,33,34]. Similarly, increasing comorbidity burden was associated with longer hospital stays. Patients with multiple chronic conditions often experience polypharmacy, reduced physiological tolerance during acute illness and greater susceptibility to treatment-related adverse effects, all of which complicate recovery. Moreover, hospital admission decisions are not always driven solely by medical factors; social circumstances and functional decline may render home care impossible, thereby prolonging hospitalization. Prior research has shown that coexisting chronic diseases significantly complicate the recovery process and contribute to extended LOS [35,36].

Regarding the relationship between malnutrition screening tools and LOS, patients classified as high-risk according to the NRS-2002 were found to have a significantly longer LOS independently. In contrast, neither the MUST nor the SGA classifications demonstrated a statistically significant effect on LOS. This finding may be explained by the structural characteristics of the NRS-2002. Unlike tools that rely primarily on chronic indicators of nutritional depletion such as anthropometric measurements or recent weight loss, the NRS-2002 provides a more comprehensive assessment by also incorporating disease severity. Therefore, particularly in acute care environments such as the emergency department, where illness burden and metabolic stress are prominent, the NRS-2002 may better capture overall clinical complexity and more sensitively identify patients who require prolonged hospitalization.

Consistent with our results, several cohort studies have reported that the NRS-2002 performs strongly in predicting extended LOS and complication risk [37,38]. On the other hand, the MUST is a more practical tool primarily based on BMI, recent weight loss and the acute disease effect. However, in geriatric emergency department patients, where acute physiological deterioration often predominates, the MUST may classify fewer individuals as high-risk and may therefore show a weaker association with complex in-hospital outcomes such as LOS. Similarly, the SGA is a more subjective instrument incorporating clinical judgment based on patient history and physical examination. Its reliance on clinician assessment may lead to greater variability in standardization compared with the MUST or the NRS-2002, which could have contributed to the borderline statistical impact of the SGA on LOS in our cohort.

This study highlights the critical roles of age, nutritional status, frailty and comorbidity burden in shaping clinical trajectories among geriatric patients presenting to the emergency department. Our findings demonstrate that chronological age alone is not a sufficient risk marker; rather, the patient’s biological and functional reserve appears to be the primary determinant of acute outcomes. In clinical practice, malnutrition screening tools may have limited utility in predicting the immediate need for hospital admission when used in isolation. However, when integrated with frailty assessment (CFS), comorbidity burden (CCI) and acute physiological deterioration scores such as NEWS2, they may contribute to a more comprehensive and holistic risk stratification approach. Importantly, while malnutrition screening instruments did not independently determine hospital admission, they may still hold prognostic relevance for in-hospital outcomes, particularly length of stay. Among the evaluated tools, the NRS-2002 emerged as the most clinically informative in the emergency department setting. By incorporating acute illness severity into its scoring system, the NRS-2002 identified the highest prevalence of nutritional risk (41.5%) and was the only instrument that independently predicted prolonged hospitalization (LOS).

Taken together, these findings support the clinical importance of malnutrition risk assessment in geriatric emergency populations and suggest that the NRS-2002 may represent a more sensitive tool for anticipating in-hospital trajectories in this vulnerable group. Beyond risk stratification, early recognition of nutritional vulnerability in older ED patients may facilitate timely nutritional support and individualized care planning. Recent studies have highlighted the potential role of geriatric specific nutritional formulations and targeted nutritional interventions in improving nutritional intake and functional outcomes among older adults at risk of malnutrition [39]. Therefore, identification of high-risk patients in the ED setting may represent an important first step toward comprehensive geriatric nutritional management.

### Limitations

Several limitations of this study should be acknowledged. First, although the study was conducted prospectively, its single-center design may limit the generalizability of the findings to other emergency department settings and healthcare systems. Second, while multivariable models were adjusted for clinically relevant covariates, residual confounding cannot be fully excluded, particularly with respect to unmeasured factors such as socioeconomic status, baseline functional capacity, or prior nutritional interventions. In some non-ambulatory or clinically unstable patients, height and weight were obtained from patient or caregiver report or from prior medical records. These data may be subject to recall error and measurement bias. In addition, the NRS-2002 is not a purely nutritional instrument, as it incorporates age and disease severity, which may have introduced overlap with frailty and acute illness measures included in the multivariable models. Nutritional risk was assessed only at a single time point upon presentation, and changes in nutritional status during hospitalization were not systematically monitored. Finally, clinical outcomes were limited to hospital admission and length of stay, whereas longer-term endpoints such as post-discharge mortality, readmissions, or functional decline were not evaluated. Future studies with larger sample sizes, multicenter designs, and extended follow up are warranted to validate these findings and to determine whether early nutritional screening and targeted interventions can improve clinical outcomes in geriatric emergency populations.

## 5. Conclusions

In conclusion, this prospective study addressed the clinical need to clarify whether malnutrition screening tools provide meaningful information during the early evaluation of older adults in the emergency department. Although nutritional risk was common and varied substantially according to the screening tool used, the MUST, the NRS-2002, and the SGA did not independently predict immediate hospital admission after adjustment for acute illness severity, frailty, and comorbidity burden. This suggests that ED disposition decisions in older adults are primarily driven by acute physiological status and functional reserve rather than nutritional risk alone. However, the NRS-2002 was independently associated with longer hospital length of stay, indicating that nutritional screening may still be useful for identifying patients who require more complex in-hospital care and resource planning. These findings support the integration of nutritional assessment with frailty, comorbidity, and acute severity measures in geriatric ED care. Larger, multicenter studies are needed to validate these findings and to determine whether early nutritional interventions can improve outcomes in high-risk older ED patients.

## Figures and Tables

**Table 1:** Baseline demographic and clinical characteristics of patients.

	Overall	Hospitalization (+)	Hospitalization (−)	*p* Value
*Age (years)*	76.4 ± 7.2	76.3 ± 6.9	76.8 ± 7.3	0.45
*Sex, n(%)*				0.49
Female	169 (52%)	58 (54.7%)	111 (50.7%)	
Male	156 (48%)	48 (45.3%)	108 (49.3%)	
*Weight (kg)*	73.9 ± 15.4	74.5 ± 16.9	73.7 ± 15.1	0.74
*Height (cm)*	163 ± 8.5	163 ± 8	164 ± 8	0.73
*BMI categories, n (%)*				0.15
≤18.5 kg/m^2^	15 (4.7%)	5 (4.8%)	10 (4.6%)	
18.6 24.9 kg/m^2^	87 (27.1%)	31 (29.8%)	56 (25.8%)	
25 29.9 kg/m^2^	117 (36.4%)	33 (31.7%)	84 (38.7%)	
≥30 kg/m^2^	102 (31.8%)	35 (33.7%)	67 (30.9%)	
*CCI*	5.4 ± 2.0	5.7 ± 2.0	5.3 ± 1.9	0.09
*CFS categories, n (%)*				0.74
CFS 1–3 (fit)	144 (44.3%)	44 (41.5%)	100 (45.7%)	
CFS 4 (vulnerable)	67 (20.6%)	22 (20.8%)	45 (20.5%)	
CFS ≥ 5 (frail)	114 (35.1%)	40 (37.7%)	74 (33.8%)	
*NEWS2 score*	7.3 ± 2.0	7.7 ± 2.2	7.1 ± 1.9	**0.02**

CFS: Clinical Frailty Scale, CCI: Charlson Comorbidity Index, NEWS2 score: National Early Warning Score 2.

**Table 2:** Agreement between malnutrition screening tools.

	Agreement (%)	Cohen’s κ	PPA (%)	NPA (%)
MUST (≥2) vs. NRS-2002 (≥3)	73.8	0.41	55.0	81.6
MUST (≥2) vs. SGA (B/C)	87.1	0.60	67.7	91.9
NRS-2002 (≥3) vs. SGA (B/C)	78.2	0.52	66.4	83.8

MUST: Malnutrition Universal Screening Tool, NRS-2002: Nutritional Risk Screening 2002, SGA: Subjective Global Assessment.

**Table 3:** Association between malnutrition screening tools and hospital admission.

		Hospital Admission (+)	Hospital Admission (−)	*p* Value
MUST risk	Low-risk	85 (80.2%)	186 (84.9%)	0.282
	High-risk	21 (19.8%)	33 (15.1%)	
NRS-2002 risk	Low-risk	58 (54.7%)	132 (60.3%)	0.341
	High-risk	48 (45.3%)	87 (39.7%)	
SGA class	A (well-nourished)	81 (76.4%)	168 (76.7%)	0.953
	B/C (malnourished)	25 (23.6%)	51 (23.3%)	

MUST: Malnutrition Universal Screening Tool, NRS-2002: Nutritional Risk Screening 2002, SGA: Subjective Global Assessment.

**Table 4:** Multivariable logistic regression analysis of factors associated with hospital admission.

	Model A MUST			Model B NRS-2002			Model C SGA		
	OR	95% CI	*p*	OR	95% CI	*p*	OR	95% CI	*p*
Age	0.88	0.83–0.95	<0.001	0.88	0.83–0.95	<0.001	0.88	0.82–0.94	<0.001
Sex	0.81	0.49–1.35	0.41	0.82	0.49–1.36	0.43	0.82	0.49–1.36	0.44
CFS	1.70	1.26–2.29	0.001	1.71	1.27–2.30	<0.001	1.74	1.29–2.34	<0.001
NEWS2 score	1.21	1.07–1.37	0.003	1.21	1.07–1.37	0.003	1.21	1.07–1.37	0.002
CCI	1.13	0.99–1.28	0.06	1.13	1.00–1.28	0.06	1.14	1.00–1.30	0.04
MUST risk (high vs. low)	0.94	0.48–1.85	0.84						
NRS-2002 risk (high vs. low)				0.89	0.52–1.51	0.65			
SGA class (B/C vs. A)							0.69	0.37–1.29	0.24

CFS: Clinical Frailty Scale, NEWS2 score: National Early Warning Score 2, CCI: Charlson Comorbidity Index, MUST: Malnutrition Universal Screening Tool, NRS-2002: Nutritional Risk Screening 2002, SGA: Subjective Global Assessment; Multivariable logistic regression analyses were performed using hospital admission as the dependent variable. Each malnutrition screening tool (MUST, NRS-2002, and SGA) was entered separately into an identical base model to avoid multicollinearity.

**Table 5:** Association between malnutrition screening tools and hospital length of stay.

	Model A MUST			Model B NRS-2002			Model C SGA		
	*β*	95% CI	*p*	*β*	95% CI	*p*	*β*	95% CI	*p*
Age	−0.05	−0.10 to −0.01	0.02	−0.05	−0.10 to −0.01	0.01	−0.05	−0.09 to −0.01	0.02
Sex	−0.06	−0.43 to 0.29	0.70	−0.16	−0.52 to 0.19	0.37	−0.06	−0.42 to 0.29	0.73
CFS	0.22	0.01–0.42	0.03	0.18	−0.01 to 0.38	0.07	0.20	0.01–0.41	0.04
NEWS2 score	0.03	−0.04 to 0.11	0.36	0.04	−0.03 to 0.12	0.22	0.03	−0.04 to 0.11	0.42
CCI	0.11	0.02–0.20	0.01	0.11	0.02–0.20	0.01	0.10	0.01–0.19	0.02
MUST risk (high vs. low)	0.16	−0.31 to 0.63	0.49	-	-	-	-	-	-
NRS-2002 risk (high vs. low)	-	-	-	0.47	0.10–0.85	0.01	-	-	-
SGA class (B/C vs. A)	-	-	-	-	-	-	0.38	−0.05 to 0.82	0.08

CFS: Clinical Frailty Scale, CCI: Charlson Comorbidity Index, NEWS2 score: National Early Warning Score 2 MUST: Malnutrition Universal Screening Tool, NRS-2002: Nutritional Risk Screening 2002, SGA: Subjective Global Assessment. Multivariable linear regression analyses were performed using hospital length of stay as the dependent variable. Each malnutrition screening tool (MUST, NRS-2002, and SGA) was entered separately into an identical base model to avoid multicollinearity.

## Data Availability

The data presented in this study are available on request from the corresponding author. The data are not publicly available due to ethical and privacy restrictions.
